# MicroRNA expression profiling identifies miR-31-5p/3p as associated with time to progression in wild-type RAS metastatic colorectal cancer treated with cetuximab

**DOI:** 10.18632/oncotarget.5735

**Published:** 2015-10-20

**Authors:** Jitka Mlcochova, Petra Faltejskova-Vychytilova, Manuela Ferracin, Barbara Zagatti, Lenka Radova, Marek Svoboda, Radim Nemecek, Stanislav John, Igor Kiss, Rostislav Vyzula, Massimo Negrini, Ondrej Slaby

**Affiliations:** ^1^ Central European Institute of Technology, Masaryk University, Brno, Czech Republic; ^2^ Masaryk Memorial Cancer Institute, Department of Comprehensive Cancer Care, Masaryk University, Faculty of Medicine, Brno, Czech Republic; ^3^ Department of Morphology, Surgery and Experimental Medicine, University of Ferrara, Ferrara, Italy; ^4^ Department of Oncology and Radiotherapy, Faculty Hospital Hradec Kralove, Hradec Kralove, Czech Republic

**Keywords:** microRNA, metastatic colorectal cancer, EGFR, cetuximab, panitumumab

## Abstract

The aim of our study was to investigate whether microRNAs (miRNAs) could serve as predictive biomarkers to anti-EGFR therapy (cetuximab, panitumumab) in patients with RAS wild-type (wt-RAS) metastatic colorectal cancer (mCRC). Historical cohort of 93 patients with mCRC (2006–2009) was included and further divided into exploratory and validation cohorts. MiRNAs expression profiling was performed on the exploratory cohort of 41 wt-KRAS mCRC patients treated with cetuximab to identify miRNAs associated with time to progression (TTP). The validation was performed on two independent cohorts: 28 patients of wt-RAS mCRC treated with cetuximab and 24 patients of wt-RAS mCRC treated with panitumumab. We identified 9 miRNAs with significantly different expression between responders and non-responders to cetuximab therapy (*P* ≤ 0.01). These 9 miRNAs were further evaluated in two independent cohorts of patients and miR-31-3p (*P* < 0.001) and miR-31-5p (*P* < 0.001) were successfully confirmed as strongly associated with TTP in wt-RAS mCRC patients treated with cetuximab but not panitumumab. When evaluated on the complete cohort of cetuximab patients (*N* = 69), miR-31-3p (HR, 5.10; 95% CI, 2.52–10.32; *P* < 0.001) and miR-31-5p (HR, 4.80; 95% CI, 2.50–9.24; *P* < 0.001) were correlated with TTP on the comparable level of significance. There was no difference in miR-31-5p/3p expression levels in RAS mutated and wild-type tumor samples. MiR-31-5p/3p are promising predictive biomarkers of cetuximab response in wt-RAS mCRC patients.

## INTRODUCTION

Colorectal cancer (CRC) represents one of the most frequent malignant neoplasms and a leading cause of cancer related deaths in developed world. The global incidence is about 1.23 million, which is 9.7% of all cancers diagnosed worldwide [1]. Whereas 5-year survival is demonstrated in 90% patients with colorectal cancers diagnosed at an early stage, in metastic disease it is only 12% of patients who have the 5-year survival. Worth of attention is also the fact that only 39% of colorectal cancers are diagnosed at an early, localized stage, mainly because of underuse of screening options [2].

New therapeutic options for metastatic colorectal cancer (mCRC) patients, including therapy with cetuximab and panitumumab antibodies targeting epidermal growth factor receptor (EGFR), have improved patient survival. Anti-EGFR therapy was historically indicated only to mCRC patients with *KRAS* wild-type (wt-KRAS) tumors, whereas these tumors presents approximately 60% of all mCRC cases [3]. However, only 35–40% of these wt-KRAS patients have clinical benefit from anti-EGFR treatment [4]. To avoid exposing of non-responding patients to ineffective, possibly harmful and expensive therapy, great effort has been made to identify new predictive biomarkers of anti-EGFR monoclonal antibodies and finally *NRAS* and novel *KRAS* mutations were identified to be also correlated with lack of response [5, 6]. Introduction of testing for *NRAS* and rare *KRAS* mutations into clinical routine increased power of response prediction, but frequency of these mutations is not high enough to improve overall response rate in mCRC as requested and there is still a large proportion of patients who do not receive benefit from this treatment [5].

MicroRNAs (miRNAs), short 18 to 25 nucleotides long, non-coding single stranded RNAs, represent regulatory network that regulate more than half of all human coding genes on post-transcription level. They are implicated in cancer biology and act as oncogenes or tumour-suppressor and their deregulation can lead to the development of a wide range of solid tumors including CRC. Antibody immune responses and EGFR pathway and its signaling components were shown to be directly regulated by miRNAs [7]. There are also two recent reports indicating involvement of miRNAs in sensitivity of mCRC to anti-EGFR therapy [8, 9].

The aim of this study was to identify and validate miRNAs whose expression could help to predict time to progression (TTP) and response to cetuximab and/or panitumumab in wt-RAS mCRC patients.

## RESULTS

### MiRNA signature associated with response to cetuximab (exploratory cohort)

**Figure 1:**
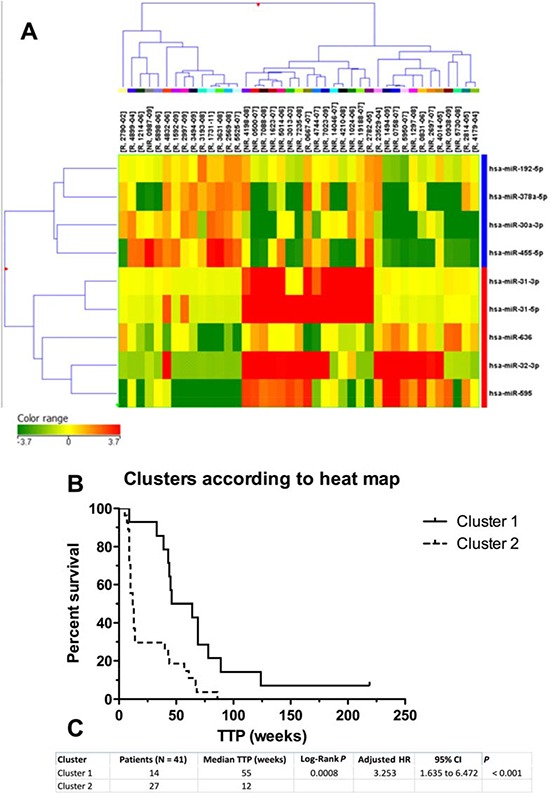
Hierarchical cluster representation of miRNAs differentially expressed in cetuximab resistance **A.** Cluster analysis groups samples and genes according to expression similarity. Genes are in rows, samples in columns. The colors of the genes represented on the heatmap correspond to the values normalized on miRNA average expression across all samples (see colorbar); up-regulated miRNAs are in red, down-regulated miRNAs in green. Downregulated genes are marked in blue, upregulated in red. **B.** Kaplan-Meier survival curves of patients treated with cetuximab clustered on the basis of miRNA expression pattern. **C.** Differences in time to progression between two clusters of cetuximab-treated patients.

Nine miRNAs are differentially expressed in responders (R) and non-responders (NR) to cetuximab therapy. To identify miRNAs with the significantly different expression in FFPE tumor samples of non-responders (TTP shorter than 25 weeks) and responders (TTP longer than 25 weeks), we analysed expression profiles of 723 miRNAs in 20 samples from non-responders and 21 samples from responders to cetuximab (Table 1). Using criterion *P* ≤ 0.01 at moderated *t*-test we identified 9 miRNAs that were used for cluster analysis (miR-31-5p, miR-31-3p, miR-192-5p, miR-378a-5p, miR-30a-3p, miR-455-5p, miR-636, miR-32-3p, miR-595). Based on a cluster analysis, Kaplan-Meier survival curves were evaluated comparing TTP between patients in the cluster 1 (*N* = 14) and cluster 2 (*N* = 27). Median TTP in cluster 1 was 55 weeks, median TTP in cluster 2 was 12 weeks (Figure 1). The most significantly upregulated miRNAs in non-responders compared to responders (*P* ≤ 0. 01) were miR-31-5p (Fold Change, FC = 14.746), miR-31-3p (FC = 6.747), miR-595 (FC = 5.555), miR-636 (FC = 2.95) and miR-32-3p (FC = 6.732). Oppositely, the most significantly downregulated miRNAs in NR/R (*P* ≤ 0. 01) were miR 378a-5p (FC = 6.689), miR-192-5p (FC = 1.881), miR-455-5p (FC = 5.019) and miR-30a-3p (FC = 4.499) (Table 2).

**Table 1:** Clinical characteristics of patients

Patient characteristics	Exploratory set	Validation set 1	Validation set 2
	*N* = 41	*N* = 28	*N* = 24
Gender			
Male	30 (73.2%)	17 (60.7%)	15 (62.5%)
Female	11 (26.8%)	11 (39.3%)	9 (37.5%)
Age			
Median	54	61	68
Range	54,8 (31–72)	60 (48–76)	66 (45–81)
Chemotherapy regimen			
Cetuximab irinotecan	38	13	0
Cetuximab FOLFOX, FOLFIRI	0	1	0
Cetuximab FOLFIRI	2	12	0
Cetuximab FOLFOX	0	1	0
Cetuximab deGramont	0	1	0
Cetuximab XELIRI	1	0	0
Panitumumab	0	0	22
Panitumumab FOLFIRI	0	0	2
Number of treatment lines before anti-EGFR therapy			
Median	3	2	1
Range	(2–5)	(2–4)	(1–3)
Response according to RECIST criteria			
Complete response	6	0	1
Partial response	13	6	4
Stable disease	2	8	6
Progressive disease	20	14	13

**Table 2:** MicroRNAs differentially expressed (*P* ≤ 0.01) between non-responders and responders to cetuximab (exploratory set of samples, *N* = 41)

MicroRNA	*P* value	Fold change	Regulation NR/R	NR (average)	R (average)	Chromosome	Mirbase accession
miR-31-5p	0.0002	14.75	up	3.46	0.23	chr9	MIMAT0000089
miR-31-3p	0.0018	6.75	up	1.19	0.18	chr9	MIMAT0004504
miR-595	0.0054	5.56	up	3.83	0.69	chr7	MIMAT0003263
miR-32-3p	0.0058	6.73	up	2.79	0.42	chr9	MIMAT0004505
miR-636	0.0093	2.95	up	5.15	1.74	chr17	MIMAT0003306
miR-378a-5p	0.0016	6.69	down	0.56	3.78	chr5	MIMAT0000731
miR-192-5p	0.0033	1.88	down	1097.67	2065.23	chr11	MIMAT0000222
miR-455-5p	0.0039	5.02	down	0.39	1.97	chr9	MIMAT0003150
miR-30a-3p	0.0061	4.50	down	0.82	3.7	chr6	MIMAT0000088

Regulation NR/R in the fourth column shows if the miRNA of interest is down or up regulated in non-responders compared to responders.

NR = non-responder to anti-EGFR therapy, R = responder to anti-EGFR therapy.

### MiR-31-5p/3p are associated with TPP in patients treated with cetuximab (validation cohort 1)

**Figure 2:**
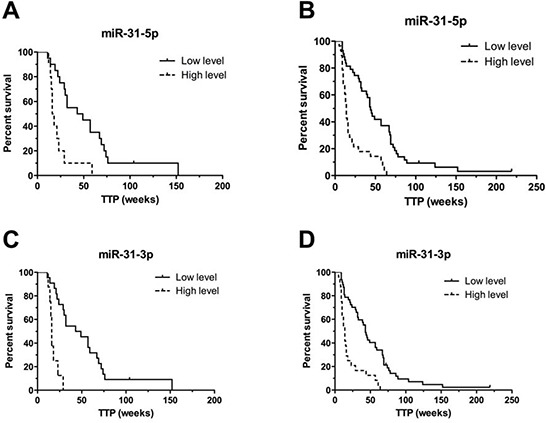
Kaplan-Meier survival curves of patients treated with cetuximab estimating TTP in weeks according to miR-31-5p and miR-31-3p expression profiles (*P* < 0.001) Patients with high expression level of relevant miRNA are illustrated by dashed line. **A, C.** performed on validation set 1 (*N* = 28), **B, D.** performed on complete set of cetuximab samples from exploratory and validation set 1 (*N* = 69).

Validation was performed on the validation cohort 1 (*N* = 28) consisted of patients treated with cetuximab. From the nine validated miRNAs, only miR-31-5p and miR-31-3p were confirmed to be significantly associated with TTP in patients treated with cetuximab (*P* ≤ 0.001) (Table 3). Based on the cut-off values of normalized miRNA expressions determined by ROC analysis, we divided patients into two groups (with low and with high miRNA expression). Patients with high-level of miR-31-5p had TTP median of 16 weeks, with low-level 49 weeks (*P* < 0.001, adjusted HR 7.369, 95% CI 2.242 to 24.219). For miR-31-3p it was 16 vs. 49 weeks (*P* < 0.001, adjusted HR 35.051, 95% CI 6.887 to 178.412) (Table 3), (Figure 2). MiR-31-5p/3p showed strong association with response to cetuximab therapy, also when their predictive potential was evaluated on the complete set of cetuximab patients, containing both – exploratory and validation cohort 1 patients (*N* = 69). Median TTP in the group of patients with high expression of miR-31-3p was 14 vs. 44 weeks in the group of patients with low expression of this miRNA (*P* < 0.0001, HR 5.099, 95%CI 2.520 to 10.317). Median TTP in the group of patients with high expression of miR-31-5p was 14 vs. 45 weeks in the miR-31-5p-low expression-group (*P* < 0.0001, HR 4.803, 95%CI 2.497 to 9.242), (Table 4), (Figure 2). MiR-31-5p was successfully validated also as associated to objective therapy response defined accordingly to RECIST criteria (Supplementary Table S1). When compared in FFPE tumor samples with or without mutations, miR-31-5p/3p expression levels were found not to be associated with KRAS/NRAS mutational status (*P* = 0.901 and *P* = 0.813, respectively).

**Table 3:** MicroRNAs validated on validation set 1 (*N* = 28) and their correlation with TTP (weeks)

MiRNA	Patients (*N* = 28)	Median TTP (weeks)	Log-Rank *P*	Adjusted HR	95% CI	*P*
MiR-31-3p						
Low ≤ 0.0155	21	49	< 0.0001	35.051	6.887 to 178.412	<0.001
High > 0.0155	7	16				
MiR-31-5p						
Low ≤ 0.1378	19	49	0.001	7.369	2.242 to 24.219	<0.001
High > 0.1378	9	16				
MiR-378a-5p						
Low ≤ 0.0734	15	22	0.1406	0.553	0.251 to 1.216	
High > 0.0734	13	49				
MiR-595						
Low ≤ 0.0142	8	29.5	0.8797	0.935	0.393 to 2.225	
High > 0.0142	20	31				
MiR-192						
Low ≤ 4.7538	18	31	0.6333	1.290	0.369 to 1.834	
High > 4.7538	10	40				
MiR-455						
Low ≤ 0.0753	20	37.5	0.5370	1.348	0.522 to 3.483	
High > 0.0753	8	19.5				
MiR-30a-3p						
Low ≤ 0.065	16	37.5	0.6047	1.238	0.552 to 2.777	
High > 0.065	12	22				
MiR-636*						
MiR-32*						

* *Ct values of miR-636 and miR-32 were higher than 35 and considered negative*.

**Table 4:** MiR-31-3p and miR-31-5p validated on the complete set of cetuximab samples from training set and validation set 1 (*N* = 69) and their correlation with TTP (weeks)

MiRNA	Patients (*N* = 69)	Median TTP (weeks)	Log-Rank *P*	Adjusted HR	95% CI	*P*
MiR-31-3p						
Low, ≤ 0.0155	46	44	<0.0001	5.099	2.520 to 10.317	<0.001
High, > 0.0155	23	14				
MiR-31-5p						
Low, ≤ 0.1378	42	45	<0.0001	4.803	2.497 to 9.242	<0.001
High, > 0.1378	27	14				

### MiR-31-5p/3p are not associated with TPP in patients treated with panitumumab (validation cohort 2)

To determine whether it is possible to use miRNAs associated with TTP in patients treated with cetuximab also in patients undergoing therapy with different anti-EGFR monoclonal antibody- panitumumab, independent validation was performed on the validation set 2, which is consisted only of patients treated with panitumumab (*N* = 24). The validation of miR-31-5p/3p expression levels using qRT-PCR method was performed identically to validation performed in cetuximab validation cohort 1. We observed, that there is no association between miR-31-5p/3p expression levels and response to panitumumab therapy based on different TTP intervals. Median TTP in the group with high expression of miR-31-3p was 29 vs. 20 weeks in the group with low expression of this miRNA (*P* = 0.2611, HR 0.616, 95%CI 0.264 to 1.435). Median TTP in the group with high expression of miR-31-5p was 31 vs. 20 weeks in the group with low expression of this miRNA (*P* = 0.0963, HR 0.483, 95%CI 0.205 to 1.139), (Supplementary Table S2), (Supplementary Figure S1).

### MiR-31-5p targets in CRC cells

Colorectal cancer cell lines (HCT-116, DLD-1 and HT-29) were transfected with miR-31-5p mimic and mimic negative control. The efficacy of the transfection was demonstrated by a 525 times average increase in miR-31-5p levels. Expression profiles of transfected cells were analysed (miR-31-5p mimic vs. mimic negative control) and 148 genes were found to be significantly downregulated (*P* < 0.01) and 84 genes upregulated (*P* < 0.01); (Supplementary Table S3). The involvement of these genes in the biological processes is listed in Supplementary Table S4, and their linkage to the cell signalling pathways is presented in Supplementary Table S5. (Gene Ontology, KEGG).

## DISCUSSION

By use of miRNA profiling we identified 9 miRNAs (miR-31-5p, miR-31-3p, miR-192-5p, miR-378a-5p, miR-30a-3p, miR-455-5p, miR-636, miR-32-3p, miR-595) with expression levels in FFPE tumor tissue significantly different in group of responders and non-responders to cetuximab therapy (*P* ≤ 0.01). Independent validation of these miRNAs on the validation cohort 1 (*N* = 28) confirmed ability to distinguish patients with different response to cetuximab only for miR-31-5p and miR-31-3p. When these miRNAs were evaluated in the complete cohort of cetuximab patients (*N* = 69), strong link between response to cetuximab therapy and miR-31-5p/3p was observed (*P* < 0.001). Combination of both miRNAs did not increase significance of prediction (data not shown). Expression levels of miR-31-5p in tumor tissue were significantly higher when compared to miR-31-3p (mean Ct in positive cases, 28 vs. 32) making this miRNA more reliable for diagnostic purposes. Because miR-31-5p is the leading strand of miR-31, we hypothesize, that miR-31-5p is responsible for major effects caused by miR-31 and miR-31-3p (passenger strand), which occurs in very low level is just reflecting changes in expression of miR-31 without significant functional consequences. Advantage of less expressed miR-31-3p could be its potentially higher sensitivity to modifications. But finally we suggest usage of miR-31-5p instead of −3p for potential application as predictive biomarker of response to cetuximab in mCRC patients. Although, in our study, we decided to define response on the basis of TTP interval (TTP > 25 weeks for responders, TTP < 25 for non-responders) instead of objective response evaluated by RECIST as done by other authors before (8, 13–16), in case of miR-31-5p we have successfully validated also its association with objective response (Supplementary Table S1).

When compared to very recent study published by Manceau et al. [8] and Mosakhani et al. [9], who identified miR-31-3p (not miR-31-5p) as miRNA associated with response to anti-EGFR therapy, in our study, miR-31-3p/5p were connected with response to cetuximab at higher level of significance (*P* < 0.0001). Based on miR-31-3p/5p expression levels, median TPP was 14 weeks for non-responders and 44–45 weeks for responders. This TTP interval (14 weeks) for patients with high levels of miR-31-5p/3p is comparable to median TTP in mCRC patients treated with anti-EGFR therapy carrying mutated variant of *KRAS* (patients who accordingly to recent guidelines are not indicated to receive anti-EGFR therapy), which ranges from 7.4 weeks to 3 months [13–16].

Moreover, based on miR-31-3p or miR-31-5p expression levels we were able to discriminate 34%, respectively 40% of patients with wt-RAS status who could be considered as non-responders beside RAS mutated patients. Wt-RAS status is manifested in 60% of mCRC patients [17]. The combination of RAS mutational status with miR-31-5p/3p expression levels could be in this respect powerful tool for identification of patients who are more likely to respond to cetuximab therapy. Further, we have evaluated expression levels of miR-31-5p/3p in independent recent group of *KRAS/NRAS* wild-type and *KRAS/NRAS* mutant group of mCRC patients and observed no difference between groups indicating that there is no relationship between miR-31-5p/3p and mutational status of *KRAS/NRAS*.

Based on these findings we decided to evaluate predictive potential of miR-31-5p/3p also in the validation cohort 2 consisted of mCRC patients treated with another anti-EGFR monoclonal antibody, panitumumab. Interestingly, we have found no association between miR-31-5p/3p levels and response to panitumumab, which is partially in contrast with previous studies [8, 9], which were based on mCRC patients treated with both, cetuximab or panitumumab, and patients were not stratified accordingly to specific monoclonal antibody. Mosakhani et al. [9] found significantly higher expression levels of miR-31-3p (*P* < 0.01) in tumors of RECIST-based non-responders to anti-EGFR therapy. Manceau et al. [8] found the link between miR-31-3p expression and progression-free survival (PFS) and defined formula to count PFS risk score (*P* = 0.012) applicable for both anti-EGFR monoclonal antibodies - cetuximab and panitumumab.

We are the first to show that miR-31-3p as well as miR-31-5p should not be used as a predictor of response to panitumumab. There are two potential explanations for predictive role of miR-31-5p/3p specifically for cetuximab: one associated with concomitant chemotherapy, second with different immune response linked to cetuximab and panitumumab antibodies. In our cohort, cetuximab was typically administered with concomitant chemotherapy (mainly irinotecan based regimens), whereas panitumumab was administered in monotherapy. From this perspective, is seems that miR-31-5p/3p could associated more probably with irinotecan then cetuximab response. Moreover, there is an increasing evidence describing link between chemotherapy and miRNAs regulatory network [10, 11] indicating miRNAs involvement in chemosensitivity or chemoresistance [12]. On other hand, this evidence did not show any specific associations of miR-31-5p/3p and chemosensitivity, even in case of topoisomerase inhibitors like irinotecan (10–12). In order to understand more our findings and figure out the role of miR-31-5p in response to cetuximab therapy, we performed *in vitro* study to identify mRNA targets of miR-31-5p in CRC. By use of three CRC cell lines and Affymetrix whole-genome expression profiling we found 148 genes to be significantly downregulated and 84 genes upregulated after transfection of the cell lines with miR-31-5p mimic. In consequent GeneOntology analysis, we observed that 16 of genes that are putatively targeted by miR-31-5p are involved in immune system processes, 7 genes are involved in cytokine-cytokine receptor interaction and 4 in chemokine signaling pathway. Since panitumumab is a fully human monoclonal antibody (IgG2) and is associated with different immune responses in comparison to antibody dependent cellular cytotoxicity induced by chimeric monoclonal antibody cetuximab (IgG1), we hypothesize, that specific predictive value of miR-31-5p/3p in cetuximab therapy is associated with specific immune response induced by cetuximab but not panitumumab.

Our study has also several limitations, which should be discussed. In the historical cohort of mCRC patients used for miRNA profiling (exploratory phase of the study) *NRAS* mutational status and information about rare *KRAS* mutation is not available. Unfortunately, there was no more tissue or DNA to be used for additional mutational analysis. Therefore, some of the cases analyzed in exploratory phase could have mutated RAS. Based on sequencing analysis of the independent validation cohorts we estimate approx. 5% mutated cases could be included in exploratory cohort by mistake. We believe, however, that this limitation is overcomed by the fact that *KRAS* and *NRAS* in tumors, which were planned to be enrolled in the validation cohorts, were sequenced and only *KRAS/NRAS* wild-type cases were included. Another limitation is the number of miRNAs, which were profiled by use of Agilent microarrays, which were developed on the basis of miRBase database release 10.1. The current release of miRBase is 21 and the number of miRNAs there is more than two-times higher than in 10.1. Although newly discovered miRNAs are usually expressed under very specific conditions, at very low levels, and in the majority of tissues are not present at all, based on that our profiling approach can not be considered as global. Also the sample size of validation cohorts, especially panitumumab cohort, is not sufficient to obtain significant statistical power. Further validation of the newly identified biomarkers in larger independent populations is therefore necessary.

Our study suggests that miR-31-5p/3p could serve as new biomarkers enabling identification of mCRC patients with wt-*KRAS* who are more likely to have shorter TTP and not respond to cetuximab therapy. This approach may significantly decrease (around 40% of wt-RAS mCRC patients) number of mCRC patients undergoing cetuximab therapy with no clinical benefit and enable wasteless indication of potentially effective therapy.

## MATERIALS AND METHODS

### Patient cohorts

Historical cohort of mCRC patients with available formalin fixed paraffin embedded (FFPE) tumor samples were divided into three cohorts (Table 1). Patients with mCRC involved in our study were treated with cetuximab (Masaryk Memorial Cancer Institute, Brno, Czech Republic) / panitumumab (Faculty Hospital, Hradec Kralove, Czech Republic) and received the first dose between years 2005 and 2010. Detailed information about therapeutic regimens is summarized in Table 1. In the exploratory cohort (*N* = 41), *KRAS* mutational status was routinely tested by use of TheraScreen:K-RAS mutation kit (DXS Diagnostic Innovations) and therefore information about *NRAS* mutational status and rare *KRAS* mutations is not known. Patients with time to progression (TTP) longer than 25 weeks were considered as responders and patients with TTP shorter than 25 weeks as non-responders to anti-EGFR therapy. Based on this criterion twenty-one patients were responders and twenty patients non-responders to cetuximab and these to groups were compared in miRNA profiling study. For the independent validation were used only cases proved to have wild-type *KRAS* and *NRAS* genes, which was tested by Illumina Tumor TruSight Sequencing Panel (Illumina Inc., USA) and Illumina MiSeq Sequencing System accordingly to manufacturer's recommendations. Independent validation cohort 1 (*N* = 28) consisted of 16 responders and 12 non-responders to cetuximab therapy and was used for the validation of results reached in exploratory phase of the study. Subsequently, samples from exploratory and validation cohort 1 were analyzed together (*N* = 69) to evaluate predictive potential of miRNAs, which were confirmed on the validation set 1 to be associated with therapy response. Validation cohort 2 (*N* = 24) consisted of 14 non-responders and 10 responders to panitumumab therapy. To evaluate correlation between RAS mutational status and expression levels of novel miRNA biomarkers another 10 RAS mutated and 30 RAS wild-type historical mCRC tumors were used in the study (Masaryk Memorial Cancer Institute, Brno, Czech Republic) to enable these supportive data for our concept. Informed consent approved by the local Ethical Committee was obtained from each patient before the treatment. Clinical data were obtained from the patient's medical records.

### FFPE sample processing and RNA isolation

The tissue samples were surgically resected and fixed in formalin and paraffin embedded. Samples were digested with Proteinase K at 55°C overnight and then total RNA enriched with miRNAs was isolated using mirVana miRNA Isolation Kit (Ambion, Austin, Texas, USA). RNA concentration and purity were determined spectrophotometrically (A260:A280 > 2.0; A260:A230 > 1.8) using Nanodrop ND-1000 (Thermo Scientific, USA).

### MiRNA profiling

MiRNA profiling based on direct hybridization without sample amplification was performed on the set of 21 responders and 20 non-responders FFPE tissue samples using Agilent MiRNA MicroArrays (#G4470B; Agilent Technologies, Santa Clara, CA, USA). These microarrays consist of 60-mer DNA probes synthesized *in situ* and contain 15.000 features which represent 723 human miRNAs, sourced from the Sanger miRBASE database (Release 10.1). RNA labeling and hybridization were performed in accordance to manufacturer's indications. Agilent scanner and the Feature Extraction 10.5 software (Agilent Technologies) were used to obtain the microarray raw-data. Data transformation was applied to set all the negative raw values at 1.0, followed by a Quantile normalization and log2 transformation. Filters on gene expression were used to keep only the miRNAs expressed in at least one sample.

### Validation assays: reverse transcription and qRT-PCR

Data obtained from miRNA profiling were validated by Real Time PCR that consists two steps : reverse transcription and quantitative Real-Time PCR amplification. cDNA was synthesized using TaqMan® MicroRNA Reverse Transcription kit with stem-loop RT-microRNA-specific primers (#4366597, Applied Biosystems, USA) according to the TaqMan MicroRNA Assay protocol (Applied Biosystems). Real Time PCR was performed using the Applied Biosystem 7500 Sequence Deteciton System, TaqMan® Universal Master Mix (#4440040, Applied Biosystems, USA) and TaqMan® microRNA Assays (Applied Biosystems, USA) according to the manufacturer's protocol. Threshold cycle (CT) values were calculated by SDS 2.0.1 software (Applied Biosystems, CA, USA) using the manual threshold settings 0.2. MiRNAs expression levels were normalized to the expression level of miR-1233, which was used as endogenous control. MiR-1233 was selected as reference gene through combination of GeneNorm and NormFinder algorithms. 2^− ΔCT^ method was applied to determine relative miRNA expression levels, where ΔCTs were calculated by following formula: ΔCT = CT_(miRNA of interest)_ − CT_(miR-1233)_.

### Cell culture and transfection with miR-31-5p mimic

Three colorectal carcinoma cell lines (HCT-116, HT-29 and DLD-1) obtained from the American Type Culture Collection (ATCC) were used for cell culture studies. The authentication of the cells has been provided using the short tandem repeat profiling method (Generi Biotech, Ltd., Czech Republic). Cell lines were maintained in Dulbecco's Modified Eagle Medium (DMEM) supplemented with Fetal Bovine serum (10%), 100 μg ml^−1^ penicillin, 100 μg ml^−1^ streptomycin, 0.1 mM nonessential amino acids, 2 mM L-glutamine, and 1 mM sodium pyruvate (all purchased from Invitrogen, Gibco, Carlsbad, CA, USA). Cell lines were incubated at 37°C with 5% CO_2_. All cell lines were transfected with miRVana miRNA (Ambion) mimic negative control or hsa-miR-31-5p miRVana miRNA mimic. Transfections were done with 2.5 μl lipofectamine RNAiMAX using 5 μM MiRNA mimic and 1000.000 HCT-116 cells, 1400.000 DLD-1 cells and 2000.000 HT-29 cells in a 6 wells plate. Cells were harvested 48 hours after transfection. QIAzol lysis Reagent (Qiagen) was used and RNA was isolated using Direct-zol™ RNA MiniPrep (Zymo Research) according to manufacturer's protocol. The efficacy of the transfection was assessed using TaqMan miRNA expression assay for miR-31-5p and RNU-48 as a reference gene.

### Whole-genome expression profiling of transfected cell lines

Whole-genome expression profiling was performed in paired samples of three studied cell lines (cell line transfected with miR-31-5p mimic and cell lines transfected with control mimic). Total RNA was purified from transfected cells by use of mirVana miRNA Isolation Kit (Ambion, Austin, Texas, USA). Total RNA purity was determined using Nanodrop ND-1000 (Thermo Scientific, USA), (A260:A280 > 2.0; A260:A230 > 2). RNA integrity was evaluated using Bioanalyzer 2100 (Agilent, USA). Two hundred and fifty ng of total RNA were used for GeneChip® Whole Transcript (WT) Expression Arrays (Affymetrix) according to manufacturer's protocol. cDNA was hybridized to GeneChip human Gene 2.0 ST (Affymetrix) at 45°C for 16 hours. Subsequently, GeneChips were washed and scanned (GeneChip® Scanner 3000 7G, Affymetrix). The whole-genome expression data, Affymetrix raw data (.cel files), were normalized using the robust multichip average (RMA) algorithm from ‘oligo’ Bioconductor package in R version 3.0.1.

### Statistical analysis

Agilent Human miRNA microarray results were analyzed using the GeneSpring GX 12 software (Agilent Technologies). Differentially expressed miRNAs were identified by comparing non-responders (*N* = 20) and responders (*N* = 21) defined in Patient cohorts. A 1.5 fold-change (FC) filter and the moderated *t*-test, with *P* < 0.01 and 10% false discovery rate (FDR), were applied. Differentially expressed genes were employed in cluster analysis, using the Pearson's correlation as a measure of similarity and centroid linkage. Microarray data are deposited at ArrayExpress database under accession number E-MTAB-3548.

Normalized miRNA expression data reached by qPCR analysis in validation phase of the study were statistically evaluated using GraphPad Prism 5.0 (GraphPad Prism Software, Inc.). Kaplan-Maier survival analysis were performed to evaluate the correlation between the normalized miRNAs expression levels and TTP of the patients treated with anti-EGFR therapy. Results with *P* ≤ 0.05 were considered as statistically significant. Cut-off values for Kaplan-Maier survival analysis were determined by ROC curve analysis, which were based on a correlation between patients response to anti-EGFR therapy and normalized expression level of each of validated miRNA. Patients with TTP longer than 25 weeks were considered responders to anti-EGFR therapy, patients with TTP shorter than 25 weeks were non-responders.

The whole-genome expression data were analysed by use of Bioconductor package in R version 3.0.1. The LIMMA package was used to identify differentially expressed genes in cell lines with increased levels of miR-31-5p. Obtained *p*-values were adjusted for multiple testing using the Benjamini-Hochberg correction. GeneOntology (http://www.geneontology.org/) was used for annotation and functional categorization of the genes identified with *P* < 0.05 adjusted as cut-off value.

## SUPPLEMENTARY FIGURE AND TABLES




