# Supplementary material for: MicroRNA expression profiling identifies miR-31-5p/3p as associated with time to progression in wild-type RAS metastatic colorectal cancer treated with cetuximab
Source: Oncotarget. 2015 Oct 20;6(36):38695–704. doi: 10.18632/oncotarget.5735 (PMC4770730; doi:10.18632/oncotarget.5735)
Supplement: Supplementary file 1 [file oncotarget-06-38695-s001.pdf]

SUPPLEMENTARY FIGURE AND TABLES

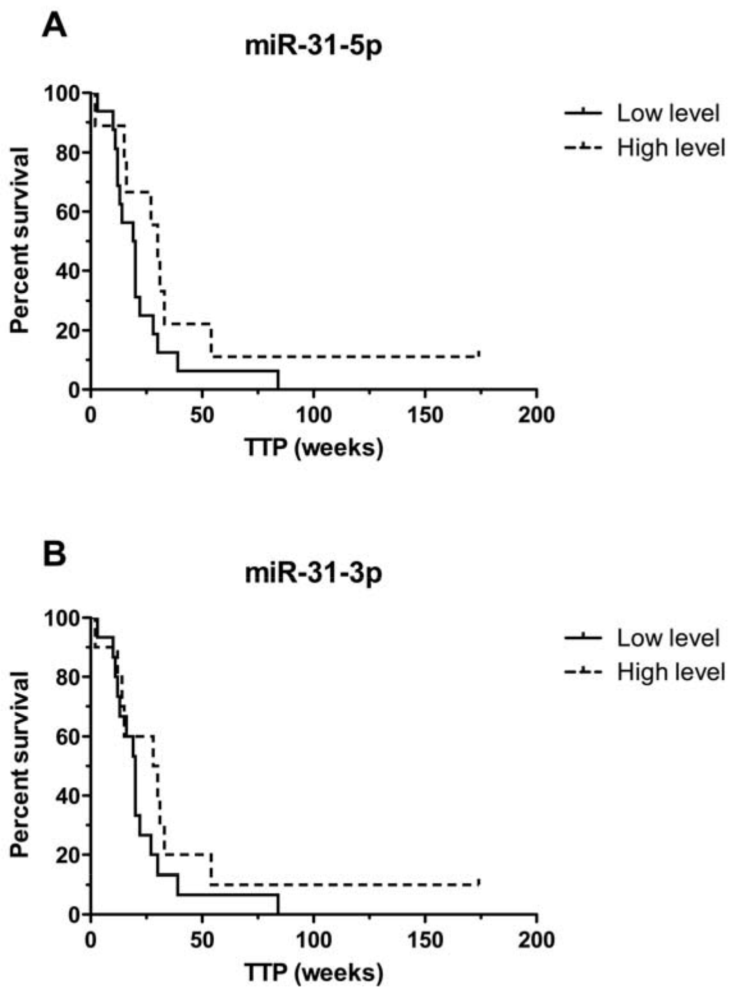

Supplementary Figure S1: Kaplan-Meier survival curves of patients treated with panitumumab estimating TTP in weeks according to miR-31-5p and miR-31-3p expression profiles (*nonsignificant*). Patients with high expression level of relevant miRNA are illustrated by dashed line. **A.** Kaplan-Meier curve estimating TTP in weeks according to miR-31-5p expression. **B.** Kaplan-Meier curve estimating TTP in weeks according to miR-31-3p expression

Supplementary Table S1: Increased expression levels of miR-31-5p are associated with response to cetuximab defined by RECIST criteria (R, responders; NR, non-responders)

Cohort of patients	Total R	Total NR	↑miR-31-5p		*P*-value
			Responders	Non-responders	
Exploratory – cetuximab	21	20	4	14	0.002
Validation 1 – cetuximab	14	14	1	8	0.0128
Exploratory + validation 1	35	34	5	22	<0.0001
Validation 2 – panitumumab	11	13	5	3	0.3905

Supplementary Table S2: MiR-31-3p and miR-31-5p validated on the validation set 2 consisted of panitumumab samples ($N = 24$) and their correlation with TTP (weeks)

MiRNA	Patients ($N = 24$)	Median TTP (weeks)	Log-Rank *P*	Adjusted HR	95% CI
MiR-31-3p					
Low, ≤0.0055	14	20	0.2611	0.616	0.264 to 1.435
High, >0.0055	10	29			
MiR-31-5p					
Low, ≤0.12	16	20	0.0963	0.483	0.205 to 1.139
High, >0.12	8	31			

Supplementary Table S3: List of genes significantly differently expressed between miR-31-5p mimic and negative control mimic ($P < 0.01$) identified in the GeneChip® Whole Transcript (WT) Expression Arrays (Affymetrix) in three colorectal cancer cell lines (HCT-116, DLD-1 and HT-29).

Supplementary Table S4: List of genes significantly differently expressed between miR-31-5p mimic and negative control mimic ($P < 0.01$) identified in the GeneChip® Whole Transcript (WT) Expression Arrays (Affymetrix) in three colorectal cancer cell lines (HCT-116, DLD-1 and HT-29) and their involvement into the biological processes (Gene ontology).

Supplementary Table S5: List of genes significantly differently expressed between miR-31-5p mimic and negative control mimic (*P* < 0.01) identified in the GeneChip® Whole Transcript (WT) Expression Arrays (Affymetrix) in three colorectal cancer cell lines (HCT-116, DLD-1 and HT-29) and their involvement in cell signalling pathways (KEGG)

Pathway name	*P*-value	Genes
Chemokine signaling pathway	0.01598	ADCY8, CXCL12, CXCR5, STAT3,
Jak-STAT signaling pathway	0.00130	IFNL1, IL7R, IL13, STAT3, OSMR,
Progesterone-mediated oocyte maturation	0.03216	ADCY8, MOS,
Protein digestion and absorption	0.02683	COL11A2, CTRB1,
Hematopoietic cell lineage	0.00521	CSF1R, ANPEP, IL7R,
Pancreatic secretion	0.00839	ADCY8, CTRB1, ATP2A3,
Cytokine-cytokine receptor interaction	0.00090	CSF1R, IFNL1, IL7R, IL13, CXCL12, CXCR5, OSMR,
Melanogenesis	0.04654	ADCY8, FZD1,
Calcium signaling pathway	0.00251	CHRM1, ADCY8, ATP2A3, P2RX5, RYR1,
Taste transduction	0.00828	ADCY8, TAS2R20,
Mucin type O-Glycan biosynthesis	0.00174	GALNT5, C1GALT1C1
