# Supplementary material for: MicroRNA expression profiling identifies miR-31-5p/3p as associated with time to progression in wild-type RAS metastatic colorectal cancer treated with cetuximab
Source: Oncotarget. 2015 Oct 20;6(36):38695–704. doi: 10.18632/oncotarget.5735 (PMC4770730; doi:10.18632/oncotarget.5735)
Supplement: Supplementary file 2 [file oncotarget-06-38695-s002.docx]

**Table S3.** List of genes significantly differently expressed between miR-31-5p mimic and negative control mimic (*P*<0.01) identified in the GeneChip® Whole Transcript (WT) Expression Arrays (Affymetrix) in three colorectal cancer cell lines (HCT-116, DLD-1 and HT-29)

| **Gene symbol** | **Pre-miR vs negative control** | **FC** |
| --- | --- | --- |
| MAGEL2 | down-regulated | 1.89 |
| RN5S304 | down-regulated | 1.6 |
| RN5S488 | down-regulated | 1.61 |
| FAM48B1,FAM48B2 | down-regulated | 1.56 |
| FAM48B1,FAM48B2 | down-regulated | 1.56 |
| C2orf48 | down-regulated | 1.61 |
| PRR23A | down-regulated | 1.53 |
| LOC100134868 | down-regulated | 1.92 |
| CALHM3 | down-regulated | 1.54 |
| PPYR1 | down-regulated | 1.53 |
| SPDYE3 | down-regulated | 1.69 |
| MIR181C | down-regulated | 1.61 |
| CSPG4P5 | down-regulated | 1.58 |
| TMEM37 | down-regulated | 1.46 |
| PNPLA5 | down-regulated | 1.48 |
| ZNF840 | down-regulated | 1.53 |
| GALNT5 | down-regulated | 1.49 |
| HES3 | down-regulated | 1.47 |
| GBGT1 | down-regulated | 1.44 |
| KBTBD13 | down-regulated | 1.53 |
| OSMR | down-regulated | 1.5 |
| CXCR5 | down-regulated | 1.44 |
| RN5S148 | down-regulated | 1.51 |
| RN5S416 | down-regulated | 1.43 |
| SEPT5-GP1BB | down-regulated | 1.45 |
| RN5S191 | down-regulated | 1.6 |
| SFMBT2 | down-regulated | 1.41 |
| KRTAP5-3 | down-regulated | 1.5 |
| STARD10-AS1 | down-regulated | 1.57 |
| LINC00477 | down-regulated | 1.4 |
| COL11A2 | down-regulated | 1.56 |
| COL11A2 | down-regulated | 1.56 |
| RARRES2 | down-regulated | 1.44 |
| HHLA2 | down-regulated | 1.42 |
| OR9Q1 | down-regulated | 1.42 |
| MOS | down-regulated | 1.43 |
| RN5S414 | down-regulated | 1.59 |
| MIR150 | down-regulated | 1.39 |
| NHLRC1 | down-regulated | 1.43 |
| LOC100131138 | down-regulated | 1.44 |
| LMOD1 | down-regulated | 1.38 |
| NTF3 | down-regulated | 1.4 |
| HGFAC | down-regulated | 1.39 |
| LMX1A | down-regulated | 1.38 |
| ENTPD8 | down-regulated | 1.44 |
| DGCR6L | down-regulated | 1.4 |
| ITIH5 | down-regulated | 1.43 |
| FAM201B,LOC100132249,LOC643395,LOC100652919 | down-regulated | 1.41 |
| HS3ST6 | down-regulated | 1.51 |
| ATP2A3 | down-regulated | 1.45 |
| TMEM98 | down-regulated | 1.41 |
| KRT33B | down-regulated | 1.41 |
| BEND5 | down-regulated | 1.4 |
| RN5S346 | down-regulated | 1.49 |
| ACTRT3 | down-regulated | 1.37 |
| RN5S413 | down-regulated | 1.44 |
| RN5S84 | down-regulated | 1.42 |
| IL17D | down-regulated | 1.49 |
| LOC440300,LOC440297 | down-regulated | 1.36 |
| C5orf60 | down-regulated | 1.4 |
| LOC642648 | down-regulated | 1.39 |
| LOC401589,LOC100129860 | down-regulated | 1.36 |
| XKR7 | down-regulated | 1.4 |
| FLJ90680 | down-regulated | 1.46 |
| PTGDR2 | down-regulated | 1.4 |
| IL13 | down-regulated | 1.36 |
| ZDHHC22 | down-regulated | 1.41 |
| NES | down-regulated | 1.36 |
| HSD3BP4 | down-regulated | 1.44 |
| LOC100288160 | down-regulated | 1.46 |
| TRANK1 | down-regulated | 1.37 |
| TBC1D2B,LOC646938 | down-regulated | 1.51 |
| PRLH | down-regulated | 1.36 |
| FZD1 | down-regulated | 1.38 |
| LOC100130071 | down-regulated | 1.55 |
| FGFBP3 | down-regulated | 1.43 |
| RN5S421 | down-regulated | 1.54 |
| ZNF697 | down-regulated | 1.4 |
| AATK-AS1 | down-regulated | 1.37 |
| LHFPL4 | down-regulated | 1.41 |
| C1orf189 | down-regulated | 1.4 |
| LOC100130744 | down-regulated | 1.38 |
| EMID1 | down-regulated | 1.41 |
| LOC100130857 | down-regulated | 1.39 |
| TMIE | down-regulated | 1.42 |
| CHRM1 | down-regulated | 1.38 |
| NINJ2 | down-regulated | 1.38 |
| TREML2 | down-regulated | 1.35 |
| ASGR1 | down-regulated | 1.38 |
| ODZ2 | down-regulated | 1.43 |
| C2CD4A | down-regulated | 1.48 |
| C20orf181 | down-regulated | 1.47 |
| LOC100129572,ARHGAP10 | down-regulated | 1.4 |
| EXD3 | down-regulated | 1.42 |
| C19orf71 | down-regulated | 1.34 |
| PPP1R2P9 | down-regulated | 1.4 |
| LOC387720 | down-regulated | 1.34 |
| LOC646513 | down-regulated | 1.34 |
| C6orf25 | down-regulated | 1.4 |
| RNY4P13 | down-regulated | 1.63 |
| C11orf16 | down-regulated | 1.35 |
| PTCHD2 | down-regulated | 1.39 |
| PRR19 | down-regulated | 1.36 |
| ZNF527 | down-regulated | 1.36 |
| OR6C76 | down-regulated | 1.34 |
| RNU1-24P | down-regulated | 1.39 |
| ANPEP | down-regulated | 1.36 |
| CXCL12 | down-regulated | 1.34 |
| SPPL2C | down-regulated | 1.41 |
| MIR187 | down-regulated | 1.48 |
| SPRED3 | down-regulated | 1.33 |
| LIM2 | down-regulated | 1.39 |
| KCNT1 | down-regulated | 1.34 |
| C1orf177 | down-regulated | 1.36 |
| TLE2 | down-regulated | 1.57 |
| HOXD10 | down-regulated | 1.32 |
| SYNE3 | down-regulated | 1.33 |
| IGHV3-72,IGHV3-73 | down-regulated | 1.35 |
| SRCRB4D | down-regulated | 1.44 |
| IGKV1D-27 | down-regulated | 1.48 |
| KIF25 | down-regulated | 1.36 |
| C1orf186,LOC100505650 | down-regulated | 1.35 |
| C6orf195 | down-regulated | 1.33 |
| NPBWR2 | down-regulated | 1.44 |
| BPIFB6 | down-regulated | 1.33 |
| CSF1R | down-regulated | 1.32 |
| P2RX5,P2RX5-TAX1BP3 | down-regulated | 1.32 |
| LAIR2 | down-regulated | 1.35 |
| LOC100129395,MLLT6 | down-regulated | 1.35 |
| LOC440700 | down-regulated | 1.33 |
| CTRB2,CTRB1 | down-regulated | 1.34 |
| MBOAT1 | down-regulated | 1.39 |
| PRDM16 | down-regulated | 1.35 |
| IL29 | down-regulated | 1.33 |
| SFTPD | down-regulated | 1.32 |
| ADCY8 | down-regulated | 1.31 |
| COL8A2 | down-regulated | 1.31 |
| LOC100506571 | down-regulated | 1.43 |
| NAT8L | down-regulated | 1.48 |
| FAM176B | down-regulated | 1.32 |
| OR2G3 | down-regulated | 1.34 |
| LOC643797 | down-regulated | 1.36 |
| RYR1 | down-regulated | 1.33 |
| WIF1 | down-regulated | 1.32 |
| DGCR5,LOC100506454,LOC100287576 | down-regulated | 1.33 |
| TRNAF15P | down-regulated | 1.34 |
| C1QL2 | down-regulated | 1.35 |
| KLHDC7B | down-regulated | 1.35 |
| TRNAP24P | up-regulated | 1.79 |
| AMZ2P1 | up-regulated | 1.82 |
| GKAP1 | up-regulated | 1.7 |
| C1GALT1C1 | up-regulated | 1.64 |
| TNFAIP3 | up-regulated | 1.59 |
| IGBP1P1 | up-regulated | 1.59 |
| NUDT13 | up-regulated | 1.54 |
| LINC00467 | up-regulated | 1.51 |
| TDG | up-regulated | 1.58 |
| C8orf37 | up-regulated | 1.6 |
| TERF1P5 | up-regulated | 1.46 |
| EIF1AY | up-regulated | 1.52 |
| ZNF673 | up-regulated | 1.48 |
| MAPKAPK5-AS1 | up-regulated | 1.55 |
| STEAP1 | up-regulated | 1.51 |
| RNU4-9P | up-regulated | 1.57 |
| LLPH | up-regulated | 1.55 |
| ZNF443 | up-regulated | 1.45 |
| CCDC113 | up-regulated | 1.58 |
| LOH12CR1 | up-regulated | 1.42 |
| ANKRD46 | up-regulated | 1.47 |
| RNU6-82 | up-regulated | 1.77 |
| MOK | up-regulated | 1.45 |
| IL7R | up-regulated | 1.43 |
| RP2 | up-regulated | 1.7 |
| PUS10 | up-regulated | 1.84 |
| CCDC30 | up-regulated | 1.45 |
| NDUFA6 | up-regulated | 1.49 |
| MED18 | up-regulated | 1.46 |
| BTG3 | up-regulated | 1.57 |
| SLC9B1,SLC9B1P2,SLC9B1P3 | up-regulated | 1.6 |
| MIR181B1 | up-regulated | 1.47 |
| RN5S195 | up-regulated | 1.87 |
| SGTB | up-regulated | 1.4 |
| CCDC62 | up-regulated | 1.47 |
| FPGT | up-regulated | 1.41 |
| PTPN20A,PTPN20B | up-regulated | 1.48 |
| EXOC6B | up-regulated | 1.48 |
| RN5S465 | up-regulated | 1.38 |
| MAP3K13 | up-regulated | 1.44 |
| CRLF3 | up-regulated | 1.58 |
| PTPN20A,PTPN20B | up-regulated | 1.46 |
| EML5 | up-regulated | 1.38 |
| RFESD | up-regulated | 1.43 |
| RN5S104 | up-regulated | 1.5 |
| YRDC | up-regulated | 1.41 |
| HINT3 | up-regulated | 1.55 |
| OSTM1 | up-regulated | 1.54 |
| CCDC59 | up-regulated | 1.43 |
| FAM72D,FAM72A,FAM72B,FAM72C | up-regulated | 1.52 |
| ENPP5 | up-regulated | 1.62 |
| JHDM1D | up-regulated | 1.48 |
| FSIP1 | up-regulated | 1.39 |
| PLA2G7 | up-regulated | 1.35 |
| C9orf72 | up-regulated | 1.68 |
| C14orf28 | up-regulated | 1.44 |
| CNGA1 | up-regulated | 1.41 |
| RNY4P7 | up-regulated | 1.36 |
| RBMY1B,RBMY1A1,RBMY1E,RBMY1D,RBMY2FP,RBMY2EP | up-regulated | 1.45 |
| RNU4-6P | up-regulated | 1.49 |
| RSL24D1P11 | up-regulated | 1.38 |
| OR4D10,OR4D11,OR4D9 | up-regulated | 1.36 |
| CEP57L1 | up-regulated | 1.51 |
| UBE2O | up-regulated | 1.38 |
| IGIP | up-regulated | 1.35 |
| PLEKHA3 | up-regulated | 1.49 |
| C9orf85 | up-regulated | 1.35 |
| FAM47DP | up-regulated | 1.33 |
| CD58 | up-regulated | 1.37 |
| PLAC8L1 | up-regulated | 1.36 |
| PGM2L1 | up-regulated | 1.82 |
| TYW5 | up-regulated | 1.63 |
| ARL3 | up-regulated | 1.35 |
| C1orf162 | up-regulated | 1.37 |
| DCAF4L1 | up-regulated | 1.34 |
| ZBTB43 | up-regulated | 1.39 |
| KIAA0040 | up-regulated | 1.38 |
| TAS2R20 | up-regulated | 1.58 |
| ST3GAL5 | up-regulated | 1.39 |
| FAM227A | up-regulated | 1.33 |
| AHI1 | up-regulated | 1.35 |
| ZBTB25 | up-regulated | 1.34 |
| STAT3 | up-regulated | 1.4 |
| DNAH6 | up-regulated | 1.33 |

**Table S4.** List of genes significantly differently expressed between miR-31-5p mimic and negative control mimic (*P*<0.01) identified in the GeneChip® Whole Transcript (WT) Expression Arrays (Affymetrix) in three colorectal cancer cell lines (HCT-116, DLD-1 and HT-29) and their involvement into the biological processes (Gene ontology).

| **Biological process** | **MiR-31-5p putative targets** |
| --- | --- |
| Reproduction | FZD1/CXCL12/STAT3/MOS |
| Metabolic process | PGM2L1/ZBTB43/FZD1/IFNL1/TYW5/TDG/KBTBD13/NES/ADCY8/NDUFA6/RARRES2/NTF3/HOXD10/COL8A2/C1GALT1C1/NHLRC1/ITIH5/ZBTB25/JHDM1D/STAT3/RP2/SFMBT2/TNFAIP3/LMX1A/COL11A2/COL11A2/CSF1R/IL13/ANPEP/AHI1/PRLH/PUS10/PLA2G7/TENM2/GBGT1/CHRM1/PRDM16/ZNF527/CTRB1/HGFAC/ZNF697/SEPT5/CRLF3/MOK/IL7R/PURA/ZDHHC22/TLE2/HES3/FPGT/SFTPD/GALNT5/KIF25/KRBOX4/PNPLA5/PPP1R2P9/ZNF443/CCDC59/HS3ST6/MBOAT1/ST3GAL5/ENTPD8/MAP3K13/SPRED3 |
| Cell killing | IL7R |
| Immune system process | IFNL1/CXCL12/ADCY8/RARRES2/TNFAIP3/CSF1R/IL13/CXCR5/OSTM1/PLA2G7/PRDM16/IL7R/SFTPD/PTGDR2/TREML2/CD58 |
| Growth | STAT3/LMX1A/CRLF3/IL7R/NINJ2 |
| Cellular process | P2RX5/ZBTB43/FZD1/ATP2A3/GKAP1/NPBWR2/IFNL1/TYW5/TDG/KBTBD13/CXCL12/NES/OSMR/ENPP5/ADCY8/NDUFA6/RARRES2/NTF3/HOXD10/COL8A2/C1GALT1C1/NHLRC1/ZBTB25/JHDM1D/STAT3/RP2/SFMBT2/TNFAIP3/LMX1A/COL11A2/COL11A2/RYR1/CSF1R/IL13/ANPEP/BTG3/CEP57L1/AHI1/ASGR1/PRLH/CXCR5/OSTM1/PUS10/MOS/SYNE3/KCNT1/PLA2G7/TENM2/GBGT1/CHRM1/PRDM16/ZNF527/PTCHD2/CTRB1/EXOC6B/DNAH6/ZNF697/SEPT5/CRLF3/MOK/IL7R/PURA/ZDHHC22/TLE2/HES3/SFTPD/GALNT5/KIF25/NPY4R/NINJ2/PTGDR2/KRBOX4/ARL3/NAT8L/CNGA1/PPP1R2P9/TREML2/WIF1/SLC9B1/LIM2/ZNF443/CCDC59/FGFBP3/C9orf72/MBOAT1/CD58/ST3GAL5/ENTPD8/MAP3K13/SPRED3 |
| Reproductive process | FZD1/CXCL12 |
| Biological adhesion | CXCL12/COL8A2/TENM2/NINJ2/CD58 |
| Signaling | P2RX5/FZD1/GKAP1/NPBWR2/IFNL1/CXCL12/OSMR/ADCY8/NTF3/STAT3/TNFAIP3/CSF1R/IL13/AHI1/PRLH/CXCR5/TENM2/CHRM1/PRDM16/PTCHD2/SEPT5/CRLF3/MOK/IL7R/TLE2/NPY4R/PTGDR2/ARL3/NAT8L/CNGA1/PPP1R2P9/WIF1/FGFBP3/MAP3K13/SPRED3 |
| Multicellular organismal process | P2RX5/FZD1/ATP2A3/TAS2R20/IFNL1/TDG/CXCL12/NES/ADCY8/RARRES2/NTF3/HOXD10/COL8A2/JHDM1D/TMIE/STAT3/RP2/TNFAIP3/LMX1A/COL11A2/COL11A2/RYR1/CSF1R/IL13/ANPEP/AHI1/PRLH/CXCR5/OSTM1/PLA2G7/LMOD1/TENM2/CHRM1/PRDM16/CTRB1/SEPT5/IL7R/PURA/TLE2/HES3/SFTPD/NPY4R/NINJ2/ARL3/NAT8L/CNGA1/WIF1/LIM2/FGFBP3/CD58/SPRED3 |
| Developmental process | P2RX5/FZD1/IFNL1/TDG/CXCL12/NES/RARRES2/NTF3/HOXD10/COL8A2/JHDM1D/TMIE/STAT3/RP2/TNFAIP3/LMX1A/COL11A2/COL11A2/RYR1/CSF1R/IL13/ANPEP/AHI1/PRLH/CXCR5/OSTM1/SYNE3/TENM2/CHRM1/PRDM16/IL7R/PURA/TLE2/HES3/SFTPD/NINJ2/ARL3/WIF1/LIM2/SPRED3 |
| Locomotion | CXCL12/RARRES2/NTF3/LMX1A/CSF1R/CXCR5/PLA2G7/TENM2/CHRM1/DNAH6/SFTPD/PTGDR2/CD58 |
| Single-organism process | P2RX5/FZD1/ATP2A3/GKAP1/TAS2R20/NPBWR2/IFNL1/TDG/CXCL12/NES/OSMR/ENPP5/ADCY8/RARRES2/NTF3/HOXD10/COL8A2/JHDM1D/TMIE/STAT3/RP2/TNFAIP3/LMX1A/COL11A2/COL11A2/RYR1/CSF1R/IL13/ANPEP/BTG3/CEP57L1/AHI1/ASGR1/PRLH/CXCR5/OSTM1/MOS/SYNE3/KCNT1/PLA2G7/LMOD1/TENM2/CHRM1/PRDM16/PTCHD2/CALHM3/CTRB1/EXOC6B/DNAH6/SEPT5/CRLF3/MOK/IL7R/PURA/ZDHHC22/TLE2/HES3/SFTPD/KIF25/NPY4R/NINJ2/PTGDR2/ARL3/NAT8L/CNGA1/PPP1R2P9/TREML2/STEAP1/WIF1/SLC9B1/LIM2/FGFBP3/C9orf72/CD58/MAP3K13/SPRED3 |
| Rhythmic process |  |
| Positive regulation of biological process | P2RX5/FZD1/IFNL1/CXCL12/NES/OSMR/ADCY8/RARRES2/NTF3/HOXD10/NHLRC1/JHDM1D/STAT3/TNFAIP3/CSF1R/IL13/AHI1/PLA2G7/TENM2/CHRM1/PRDM16/CRLF3/IL7R/PURA/HES3/SFTPD/PTGDR2/NAT8L/WIF1/FGFBP3/MAP3K13 |
| Negative regulation of biological process | FZD1/IFNL1/TDG/CXCL12/NES/NTF3/STAT3/TNFAIP3/LMX1A/RYR1/IL13/BTG3/AHI1/YRDC/TENM2/PRDM16/CRLF3/IL7R/PURA/TLE2/HES3/SFTPD/KIF25/SPRED3 |
| Regulation of biological process | P2RX5/ZBTB43/FZD1/GKAP1/NPBWR2/IFNL1/TDG/CXCL12/NES/OSMR/ADCY8/RARRES2/NTF3/HOXD10/NHLRC1/JHDM1D/STAT3/RP2/SFMBT2/TNFAIP3/LMX1A/RYR1/CSF1R/IL13/ANPEP/BTG3/AHI1/PRLH/CXCR5/MOS/SYNE3/YRDC/PLA2G7/TENM2/CHRM1/PRDM16/ZNF527/PTCHD2/ZNF697/SEPT5/CRLF3/MOK/IL7R/PURA/TLE2/HES3/SFTPD/KIF25/NPY4R/PTGDR2/KRBOX4/ARL3/NAT8L/CNGA1/PPP1R2P9/WIF1/ZNF443/CCDC59/FGFBP3/MAP3K13/SPRED3 |
| Response to stimulus | P2RX5/FZD1/ATP2A3/GKAP1/NPBWR2/IFNL1/TDG/CXCL12/OSMR/ADCY8/NDUFA6/RARRES2/NTF3/IL17D/HOXD10/STAT3/TNFAIP3/LMX1A/RYR1/CSF1R/IL13/AHI1/ASGR1/PRLH/CXCR5/PLA2G7/TENM2/CHRM1/PRDM16/PTCHD2/CRLF3/MOK/IL7R/TLE2/SFTPD/NPY4R/NINJ2/PTGDR2/ARL3/CNGA1/PPP1R2P9/WIF1/ZNF443/FGFBP3/CD58/MAP3K13/SPRED3 |
| Localization | P2RX5/FZD1/ATP2A3/CXCL12/ADCY8/RARRES2/NTF3/STAT3/RP2/RYR1/CSF1R/IL13/CEP57L1/AHI1/ASGR1/OSTM1/SYNE3/YRDC/KCNT1/PLA2G7/CHRM1/PTCHD2/CALHM3/EXOC6B/DNAH6/SEPT5/ZDHHC22/SFTPD/ARL3/NAT8L/CNGA1/STEAP1/SLC9B1/CD58 |
| Establishment of localization | P2RX5/FZD1/ATP2A3/ADCY8/NTF3/STAT3/RP2/RYR1/CSF1R/IL13/AHI1/ASGR1/OSTM1/YRDC/KCNT1/CHRM1/PTCHD2/CALHM3/EXOC6B/SEPT5/SFTPD/ARL3/NAT8L/CNGA1/STEAP1/SLC9B1 |
| Multi-organism process | IFNL1/CXCL12/STAT3/TNFAIP3/IL13/ANPEP |
| Biological regulation | P2RX5/ZBTB43/FZD1/ATP2A3/GKAP1/NPBWR2/IFNL1/TDG/CXCL12/NES/OSMR/ADCY8/RARRES2/NTF3/HOXD10/NHLRC1/JHDM1D/STAT3/RP2/SFMBT2/TNFAIP3/LMX1A/RYR1/CSF1R/IL13/ANPEP/BTG3/CEP57L1/AHI1/PRLH/CXCR5/MOS/SYNE3/YRDC/PLA2G7/TENM2/CHRM1/PRDM16/ZNF527/PTCHD2/ZNF697/SEPT5/CRLF3/MOK/IL7R/PURA/TLE2/HES3/SFTPD/KIF25/NPY4R/PTGDR2/KRBOX4/ARL3/NAT8L/CNGA1/PPP1R2P9/STEAP1/WIF1/ZNF443/CCDC59/FGFBP3/CD58/MAP3K13/SPRED3 |
| Cellular component organization or biogenesis | FZD1/TDG/CXCL12/NES/NTF3/COL8A2/JHDM1D/RP2/TNFAIP3/LMX1A/COL11A2/COL11A2/CSF1R/CEP57L1/AHI1/SYNE3/PLA2G7/TENM2/CTRB1/CRLF3/IL7R/ZDHHC22/SFTPD/KIF25/ARL3/LIM2/C1QL2 |
